# A survey of perceptions of exposure to new technology in residents and practicing ophthalmologists

**DOI:** 10.1186/s12886-024-03378-w

**Published:** 2024-03-28

**Authors:** Elana Meer, Krista Davidson, Kristen Harmon Ingenito, Frank Brodie, Julie M. Schallhorn

**Affiliations:** 1 Department of Ophthalmology, University of California, San Francisco, 490 Illinois Street, 94158 San Francisco, CA USA; 2 Market Scope, St. Louis, MO USA; 3 Francis I Proctor Foundation for Research in Ophthalmology, University of California, San Francisco, CA USA

**Keywords:** Innovation, Exposure to new technologies, Residency training

## Abstract

**Background:**

Incorporation of the rapid advances in ophthalmologic surgical and diagnostic techniques inherent in the field poses a challenge to residency training programs. This study investigates exposure to new technologies during residency and perception of its impact on practice patterns.

**Methods:**

Ophthalmology residents at various training levels and practicing ophthalmologists who had completed their training were invited to participate in a survey study assessing exposure to various technologies in residency and in practice. Data collection occurred from December 2022 to June 2023. Descriptive statistics were performed.

**Results:**

The study received 132 unique responses, including 63 ophthalmology residents and 69 practicing ophthalmologists. 65.2% (*n* = 45) of practicing ophthalmologists and 47.6% (*n* = 30) of current residents reported discussion/training on newly developed products on the market (e.g. premium IOLS, MIGS), was “minimally discussed but not emphasized” or “not discussed at all” in residency. 55.1% (*n* = 38) of practicing ophthalmologists reported that exposure to new technologies during residency did influence types of technologies employed during practice. The majority resident physicians reported enjoying being trained on newer technology and feeling more prepared for future changes in the field (95.2%, *n* = 60) and felt that having industry partnerships in residency enhances education and training (90.5%, *n* = 57).

**Conclusions:**

Considering how to maximize exposure to newer technologies/devices during residency training is important, and may contribute to training more confident, adaptable surgeons, who are more likely to critically consider new technologies and adopt promising ones into their future clinical practice.

**Supplementary Information:**

The online version contains supplementary material available at 10.1186/s12886-024-03378-w.

## Background

Over the last several decades, advances in technology have allowed for the continued improvement and refinement of ophthalmic surgery [1]. The plethora of new surgical technologies and procedures, although promising for the field, poses a challenge for residency training. The influx of new technologies into operating rooms requires surgical educators to provide trainee supervision and graduated independence across a wider range of techniques. Establishing a balance between teaching traditional techniques and exposure to new technologies requires a thoughtful approach as well as a comprehensive understanding of how the exposure to new technology during residency influences future practice patterns [2].

Implementation of new technology in residency education has already become a significant consideration in cataract surgery [3], with integration of phacoemulsification and even femtosecond laser-assisted cataract surgery techniques into training [4]. The reported challenges and benefits associated with incorporating such newer techniques only further reinforce the importance of considering exposure to other new technologies during resident surgical training.

Although the Accreditation Council for Graduate Medical Education (ACGME) requires residents to perform a certain number of surgical procedures in predetermined areas, there are no guidelines for teaching new technologies or devices [5]. This may contribute to the challenges in transitioning from residency training to independent surgical practice [6]. In addition, the lack of specificity of the ACGME requirements provides flexibility in how each program incorporates new technology into surgical training. There may also be significant variation in the exposure residents get to novel technologies at different affiliate hospitals within each residency training program.

Although potentially integral to the training of adaptable and innovative surgeons, the difference in exposure to new technologies within a residency training program and how that influences early practice has not yet been explored. While programs may examine overall resident satisfaction and case volume at different hospitals within each institution [7], these questionnaires rarely include questions on satisfaction with exposure to particular technologies or techniques. This study sought to examine resident and attending perceptions of exposure to new technology during residency and how exposure influences practice patterns and adoption rates in practice.

## Methods

### Study enrollment and data collection

This study was conducted in accordance with the tenets stated in the Declaration of Helsinki. Prior to beginning the study, approval was obtained for all protocols from the University of California, San Francisco Institutional Review Board. Informed consent was obtained by all participants prior to agreeing to complete the survey.

In order to elicit the perception on exposure to residency during training and in practice, a survey study was conducted. With the aim of accessing a large diverse cohort of academic and private community physicians, the authors partnered with Market Scope (Des Peres, MO) to distribute the survey to attending physicians. In order to source US ophthalmologists, Market Scope invited US ophthalmologists who self-registered and were individually verified on market-scope.com (*n* = 723) to participate in the survey, eliciting 69 total unique responses (9.54% response rate). Data was collected between December 5th 2022 and June 15th 2023. United States ophthalmology residents in all years of training and practicing ophthalmologists in the United States who completed training were also included. In order to source US ophthalmology residents, program directors and coordinators from all ACGME ophthalmology residency programs were emailed with the request to forward the survey to all their residents. Ophthalmology residents were then sent a link to the survey to participate from their program directors and/or program coordinators. Only 17 total unique responses were obtained, therefore YoungMD Connect (Bryn Mawr Communications, Conshohocken, PA) was recruited to help increase resident response numbers by reaching out to residents through a variety of methods (QR code at academic meetings, social media, email blasts), with an additional 46 resident responses amounting to a total of 63 unique responses. The datasets used and/or analyzed during the current study are available from the corresponding author on reasonable request. Survey answers were de-identified prior to any data sharing or analysis, and data was protected through the Market Scope authorized data management system. Incomplete questionnaires were not registered or included in this study.

### Survey and analysis

**Fig. 1:**
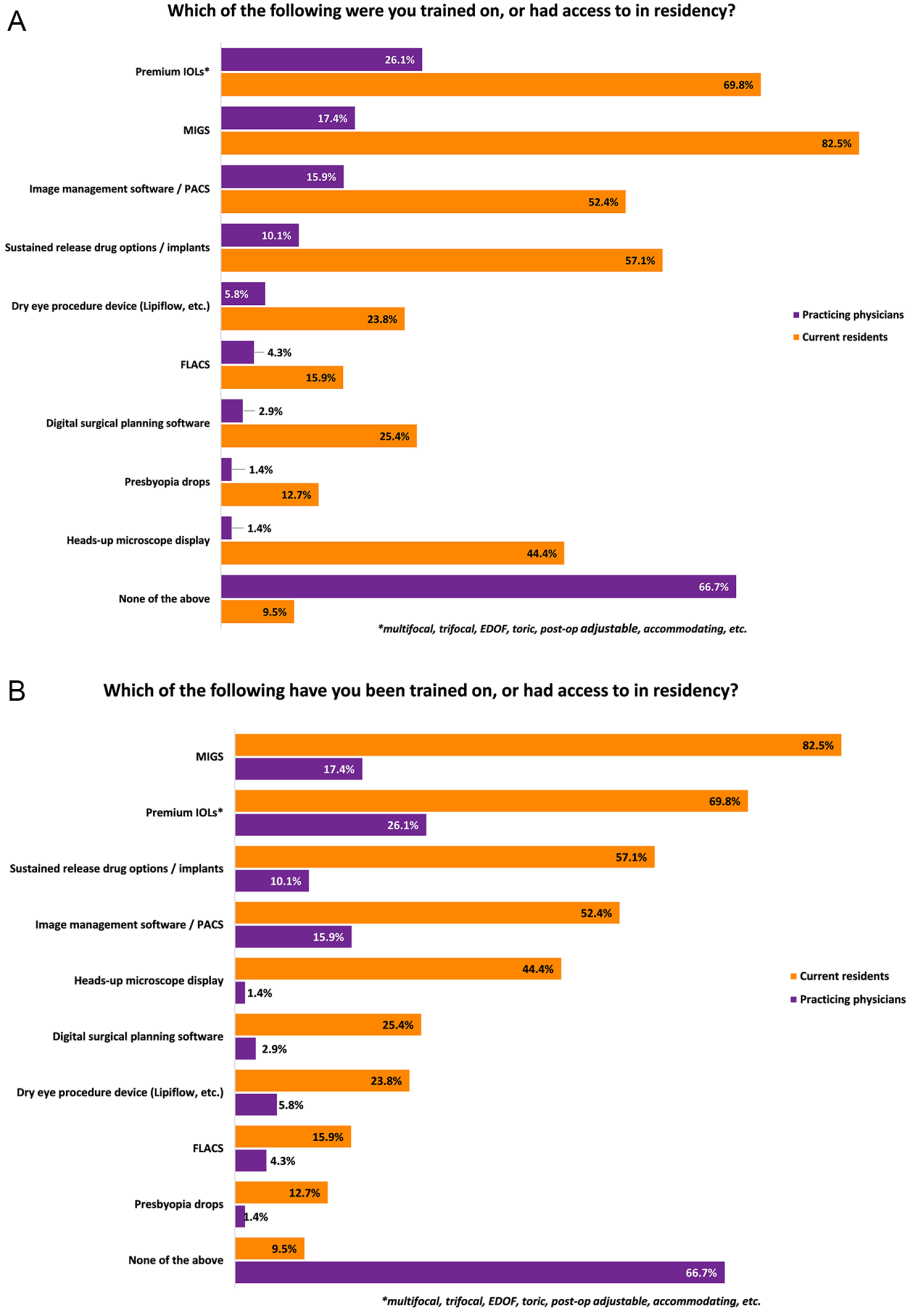
**A**. Exposure to newer technologies reported by practicing physicians. **B**. Exposure to newer technologies reported by current resident physicians

A survey was developed to assess exposure to various technologies in residency and in practice. Questions were focused on, but not limited to, soliciting feedback on practice or future practice size and structure, training received in residency, Minimally Invasive Glaucoma Surgery (MIGS) and premium intraocular lens (IOL) volumes, and current or planned use of various technologies. (Supplemental Fig. 1) Questions included year of practice, residency program (for geographic differentiation), practice setting (e.g. private practice, corporate practice, public hospital, academic center, etc.), intent to pursue fellowship, specialty, exposure to particular devices/procedures (Femtosecond Laser assisted Cataract Surgery (FLACs), digital surgical planning software, image management software/ Picture Archiving and Communication Systems (PACS), heads up microscope display, dry eye procedure device, premium IOLS, MIGS devices, presbyopia drops, sustained release drug options, etc.), perception of exposure to industry partnerships and newer technology during residency, and planned and current practice patterns for the above technologies during attending practice. (Fig. 1) Questions were primarily formatted with structured answer options in multiple choice and check relevant answer format. There was no existing peer-reviewed framework, therefore the questions were created for the purpose of this study and validated across a cohort of physicians and Marketscope team with extensive experience in developing user-friendly surveys. A summary analysis of the reported data was performed without any additional extrapolation to the overall market.

## Results

The survey was completed by 63 physician residents in a US ophthalmology program (across 42 unique programs, comprising 12.4% of the 509 US-based ophthalmology residents) and 69 physicians (9.5% of total contacted) beyond residency training between December 2022 and June 2023 (who graduated from 52 unique programs). A 36.2% (*n* = 25) majority of the practicing ophthalmologists completed residency in the 1990s. The vast majority reported being part of a group practice (76.8%, *n* = 53) (Table 1). 27% (*n* = 17) of resident respondents were on track to complete residency in 2026, 23.8% (*n* = 15) in 2025, 25.4% (*n* = 16) in 2024, 15.9% (*n* = 10) in 2023, and 6.3% (*n* = 4) in 2022.

**Table 1:** Demographics of Practicing Ophthalmologist Respondents

Primary Practice Setting	n	%
Private practice	48	69.6
Corporate practice	7	10.1
Private hospital	2	2.9
Public hospital	4	5.8
Institution/military practice	6	8.7
University	2	2.9
**Focus/specialty**	n	%
Comprehensive ophthalmology	23	33.3
Cataract and/or refractive	22	31.8
Retina	12	17.4
Glaucoma	8	11.6
Cornea and external disease	2	2.9
Oculoplastic surgery	1	1.4
Combined Glaucoma and Cataract	1	1.4

Of the practicing ophthalmologists, 81.2% (*n* = 56) were currently offering premium IOLs, and 68.1% (*n* = 47) were offering MIGS. When asked about exposure in residency, 26.1% (*n* = 18) reported being trained on premium IOLs in and 17.4% (*n* = 12) reported being trained on MIGS. When asked about the level of discussion/training participants received in their residency program on diversity of brands and manufacturers available for product selection, 56.5% (*n* = 39) percent of practicing ophthalmologists responded that this was “minimally discussed but not emphasized” or “not discussed at all.” When asked about the level of discussion/training participants received in their residency program on newly developed products on the market (premium IOLs, MIGS, etc.), 65.2% (*n* = 45) responded that this was “minimally discussed but not emphasized” or “not discussed at all.”

Practicing ophthalmologists were asked to rate their perception of exposure to newer surgical and therapeutic treatments and technologies in their own residency as compared to other programs. A total of 40.6% (*n* = 28) of respondents reported more exposure than other programs, 50.7% (*n* = 35) same exposure, and 8.7% (*n* = 6) less exposure when compared to other programs. The majority (55.1%, *n* = 38) of respondents reported that exposure to new technologies during residency did influence types of technologies employed during practice, whereas 21.7% (*n* = 15) reported that exposure did not, and 23.2% (*n* = 16) were uncertain.

Written commentary was elicited on whether respondents believed experience with technology in residency influenced the types of technology used or quantity of certain technologies in practice (Question 11 a.1, Supplementary Fig. 1). Given the low sample size, thematic analysis was performed. Those who reported minimal exposure during residency reported that less exposure made them less willing to try new technology and made them inappropriately think there were not more than one or two options for technology companies to work with. Such exemplary comments included “Less exposure makes you less willing to try new tech”, “You use what you get experience with in residency. If you do not have exposure to newer technologies, you will not feel comfortable using them.” “One brands grip on the VA system made my inappropriately think there were no other options. It was stifling.”

Those who reported same exposure and more exposure during residency when compared to other training programs provided commentary of a similar sentiment. Themes were extracted from individual comments. Respondents reported that exposure to new technologies and pharmaceuticals during residency helped define future habits and practice patterns and made them feel more adaptable and comfortable with a wide variety of techniques (e.g. various MIGS platforms and different IOLS). They also reported that greater exposure made them more open to test out new phacoemulsification machines and equipment and made them more incentivized to familiarize themselves with new cutting edge technologies. Finally, they reported that greater exposure helped them to be more adaptable surgeons with greater confidence in adopting a wide range of surgical techniques. Exemplary comments included; “Using multiple types of technology and wetlabs with industry has allowed me to be comfortable using them when in practice”, “Using them in residency allowed familiarity with it and allowed me to be less inhibited about trying other technologies”, “During residency, we were exposed to the latest phacoemulsification technology and were able to use different phaco platforms. This allowed me to decide which specific phaco platform that I wanted to use in private practice” and “We were privileged to use newer instruments and the latest surgical techniques. This experience taught me to stay ahead with these and advance patient care.”

Among resident respondents, 87.3% (*n* = 55) planned on pursuing a fellowship, and almost 39.7% (*n* = 25) planned to eventually join a private practice. 63.5% (*n* = 40) of respondents planned to treat cataracts, 46% (*n* = 29) refractive surgery, 38.1% (*n* = 24) retina, 38.1% (*n* = 24) cornea/external disease, 36.5% (*n* = 23) glaucoma, and 19% (*n* = 12) uveitis.

When asked about their perception of exposure to new technology in their own residency program versus other programs, 52.4% (*n* = 33) of residents believed that they received the same amount of exposure to newer surgical and therapeutic treatments and technologies in residency, as compared to other programs. 17.5% (*n* = 11) thought they received less exposure, 14.3% (*n* = 9) thought they received more exposure, and 15.9% (*n* = 10) percent were uncertain. When asked about partnerships with industry in terms of training and collaboration, 34.9% (*n* = 22) reported good or very good exposure and 22.2% (*n* = 14) reported poor or very poor exposure, with the rest reporting average exposure. A total of 46% (*n* = 29) reported good or very good training and availability of newer technologies and 12.7% (*n* = 8) reported poor or very poor exposure, with the rest reporting average exposure. 82.5% (*n* = 52) percent had been trained on MIGS, and 69.8% (*n* = 44) were trained on premium IOLs.

When asked about the level of discussion/training participants received in their residency program on diversity of brands and manufacturers available for product selection, 55.6% (*n* = 35) responded that this “minimally discussed but not emphasized” or “not discussed at all.” When asked about the level of discussion/training participants received in their residency program on newly developed products on the market (premium IOLs, MIGS, etc.), 47.6% (*n* = 30) responded that this was “minimally discussed but not emphasized” or “not discussed at all.” Only 4.8% (*n* = 3) reported prioritized discussion/training on diversity of brands and manufacturers available for product selection and 6.3% (*n* = 4) on newly developed products on the market (premium IOLS, MIGs, etc.).

**Fig. 2:**
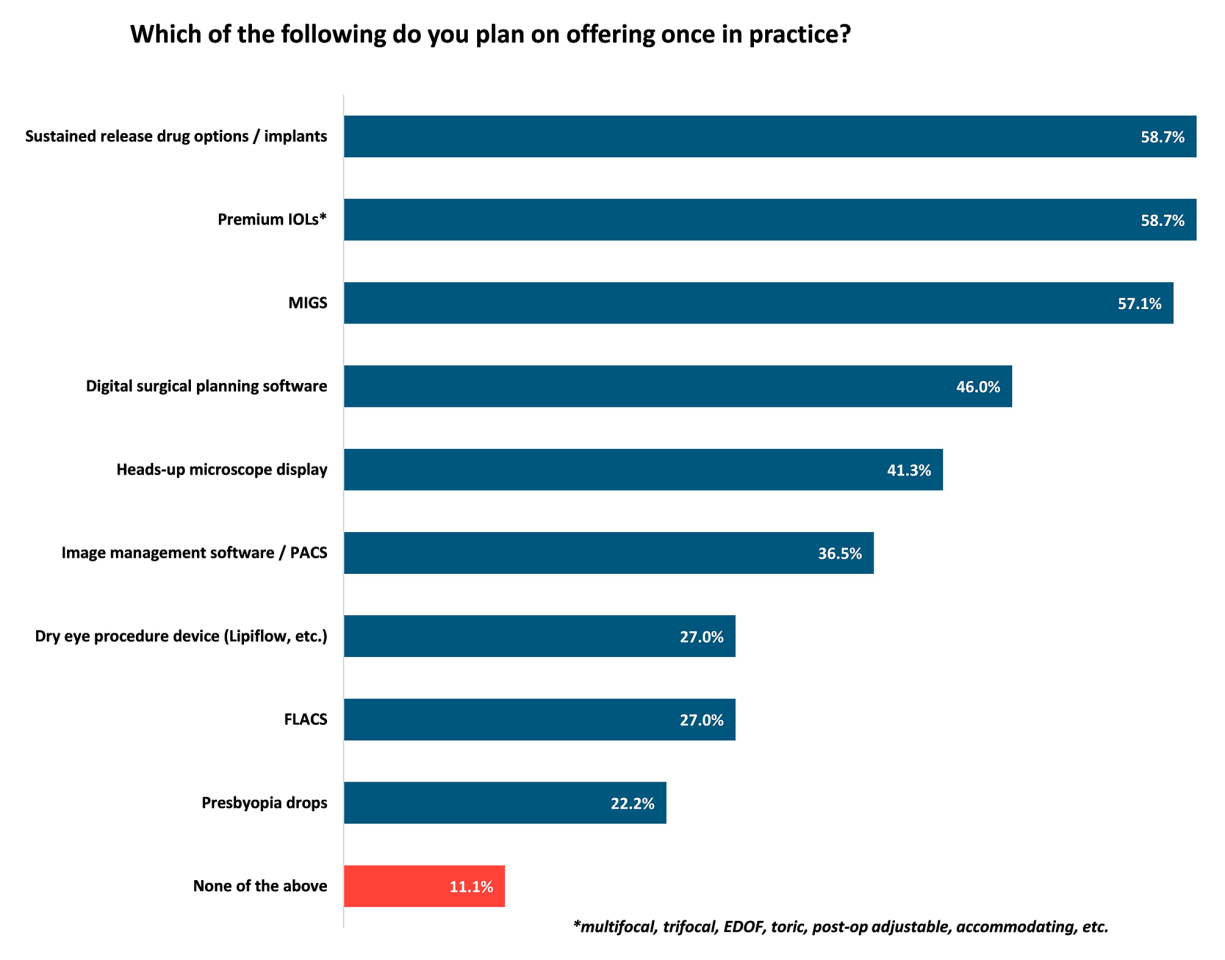
Resident response to assessment of which newer technologies they wish to offer once in practice

Responses regarding specific new technologies practicing physicians and current residents have had access to in residency are demonstrated in Fig. 1. Compared to practicing attendings, a greater percent of resident respondents reported training on or access to premium IOLS, MIGS, imaging managing software/PACS, sustained release drug options/implants, dry eye procedures, FLACS, presbyopia drops, and heads up microscope display (Fig. 1). Resident reported plans to offer certain technologies demonstrated a similar trend to the technologies residents reported training in during residency (Fig. 2).

**Fig. 3:**
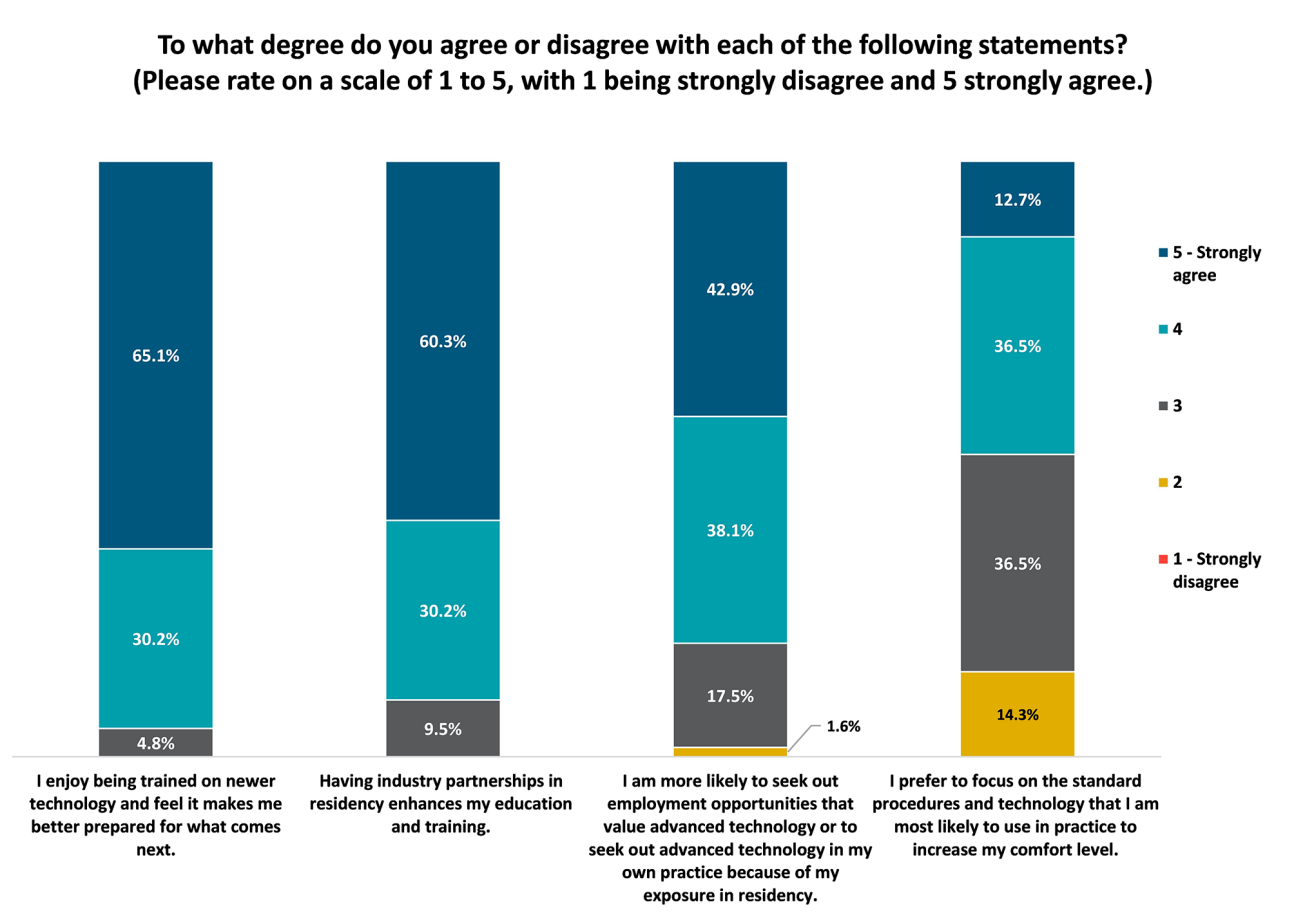
Resident Responses to assessment of importance of engaging with newer technologies and industry exposure

Overall, the vast majority resident physicians reported that they enjoyed being trained on newer technology and exposure made them feel more prepared for future changes in the field (95.2%, *n* = 60). They also reported that having industry partnerships in residency enhances education and training (90.5%, *n* = 57), and that they were more likely to seek out employment opportunities that value advanced technology because of exposure during residency (81%, *n* = 51). **(**Fig. 3) Only 12.7% (*n* = 8) strongly agreed that they would prefer to focus on the standard procedures and technology they are most likely to use in practice and to increase their comfort level **(**Fig. 3**).**

## Discussion

This study explores reported exposure to new technology in residency and perceptions of how this exposure affects and/or will affect practice patterns among current ophthalmology residents and practicing ophthalmologists in the United States. In their responses, practicing clinicians emphasized the importance of exposure to innovative technologies in residency and the majority believed exposure to new technology in residency influenced their current practice. Despite this, over half responded that discussion or training on newly developed products on the market (premium IOLS, MIGS, etc.) was minimally discussed or not discussed at all. Similarly, despite increased reported exposure to new technologies such as MIGS, premium IOLS, sustained release drug options, over half of current residents still reported that diversity of brands and manufacturers available for product selection and newly developed products on the market were minimally discussed or not discussed at all.

Combined, these findings suggest that residency programs are not perceived by trainees and graduates to have adapted to incorporate more comprehensive training newer technologies. This survey also demonstrates that residents are seeking greater training on newer technology as it makes them feel more prepared for what comes next, enhances education and training, and encourages them to further seek out advanced technology. This sentiment is supported by the corresponding responses from practicing ophthalmologists. Over half of practicing clinicians reporting that exposure and experience with technology in residency influenced the types of technology and quantity used in practice.

The written commentary elicited further sheds light on how early exposure in residency impacts the adoption of new approaches in practicing. Those with increased exposure during residency expressed a greater willingness to try new technologies and exhibit adaptability in adopting a wide range of surgical techniques. On the other hand, respondents with minimal exposure during residency felt less comfortable exploring new technologies, potentially hindering adoption of advances that improve patient care. Developing a structured approach to incorporating newer technologies/devices during residency training may contribute to training more confident, adaptable surgeons. This also may have the benefit of teaching residents to critically consider new technologies and adopt promising ones into their future clinical practice.

The challenges of incorporating new technologies into residency training has certainly been demonstrated in ophthalmology [3, 8, 9], and is present throughout all surgical fields [2, 4, 10]. Furthermore, both residents and programs hiring new graduates have perceived gaps in readiness of residency graduates for independent practice [11, 12]. With changing regulations and innovations, ophthalmology residents have reported feeling unprepared in business operations, finance, practice management, coding, advocacy [12]. Despite the importance placed on emerging technologies as a mechanism to support mentoring, precepting, and proctoring to improve transition from residency to independent practice, iterations in ACGME accreditation have been limited in their ability to foster innovation [6]. In light of this, ACGME focus groups have recognized that early experiences with new technology may serve as the basis for further exploration of innovative approaches [6, 13].

Given the challenges in striking a balance between training residents in traditional techniques and exposing them to new technologies, it is important to consider ways to incorporate exposure to new technologies without detracting from the standard ACGME case guidelines for traditional surgeries. For example, there has been concern that incorporating femtosecond laser-assisted cataract surgery into residency training may decrease manual proficiency in other parts of surgery performed by the laser, such as corneal incisions and capsulorhexis [3, 4]. Virtual reality simulators may prove beneficial to teach advanced new techniques within the bounds of academic residency training [2, 3]. Research blocks may also be utilized to develop rotations specifically focused on new technologies and techniques [10]. Structured industry partnerships may be another solution to supplement resident education without detracting from proficiency of key surgical steps.

In our survey, most respondents reported that industry partnerships in residency enhance their education and training, indicating that such collaborations can play a vital role in providing access to the latest technologies and advancements in the field. Therefore, in instances where residencies feel limited in how much exposure they can provide within the structure of a three-year program, industry partnerships may be able to fill the gaps with wet labs, didactic training sessions, and master surgeon videos to teach new techniques. In addition, greater breadth of industry exposure (rather than usage of just one or two companies’ devices), may increase physician resident confidence in critically evaluating which products they would like to incorporate into their practice to optimize patient care. Similarly, in addition to academic meetings, exposure to industry largely facilitates continued learning and training after residency, therefore establishing these relationships earlier on in training may have implications for continued training throughout practice.

This study must be considered in the context of its limitations. The small sample size of resident respondents (63 unique responses, 12.4% of US residents) may limit the generalizability of the findings. Additionally, the response rate from practicing ophthalmologists (69 unique responses) could be further improved to obtain more robust and representative results. The authors were unable to extract a view rate or participation rate, therefore the response rate is only representative of the completion rate of the online survey. This study likely suffers from response bias, as residents and practicing clinicians more interested in considering exposure to innovation and new technologies may have been more likely to complete the survey. Similarly, the study may be limited by hindsight and reporting bias, and only represents resident and practicing ophthalmologists perceived exposure during residency rather than actual exposure. In addition, given that this is a survey study, the authors may only provide reported response data, without making associations or conclusions about how exposure to new technology in residency directly affects physicians’ future practice patterns. This study also did not inquire about specific brand or manufacturer usage and exposure during residency. However, it is possible that exposure to particular brands or manufacturers during residency may influence practice patterns after residency just as it does general perception and willingness to incorporate newer technologies. In addition, further qualitative studies with more extensive commentary from the cohort of participants who reported being uncertain about the effect of exposure to newer technologies in residency may be revealing. Further research with larger sample sizes and longitudinal follow-up could provide more comprehensive data to support the conclusions and recommendations of this study. In addition, future studies would benefit from implementing a structured program to optimize exposure to new technologies, incorporating both residency program teaching and industry partnerships and prospectively measuring the impact on future practice patterns with comparisons to programs without structured innovation programs.

In conclusion, this paper draws attention to the challenges and opportunities posed by the integration of new technologies into ophthalmology residency training. It underscores the need for standardization in training pathways, ensuring a comprehensive understanding of resident exposure to new technologies, and its potential impact on their future practice patterns. The findings highlight the importance of incorporating exposure to new technologies during residency to enhance residents’ adaptability, innovation, and confidence in adopting cutting-edge surgical techniques. The insights provided by this study can guide program directors and educators in designing effective residency training programs that prepare ophthalmologists for the evolving landscape of ophthalmic surgery.

### Electronic supplementary material

Below is the link to the electronic supplementary material.

Supplementary Material 1

## Data Availability

No datasets were generated or analysed during the current study.
