# Supplementary material for: A survey of perceptions of exposure to new technology in residents and practicing ophthalmologists
Source: BMC Ophthalmol. 2024 Mar 28;24:142. doi: 10.1186/s12886-024-03378-w (PMC10976830; doi:10.1186/s12886-024-03378-w)
Supplement: Supplementary file 1 — Supplementary Material 1 [file 12886_2024_3378_MOESM1_ESM.pdf]

Market Scope®

Innovation in Residency

Introduction & Demographics

1. In what year did/will you complete residency?

2. Which residency program did/do you attend?

3. Do you plan on pursuing a fellowship? (Residents)
    a. Yes
    b. No
    c. Undecided

4. What are your plans for practicing once you complete your residency or fellowship? (Residents)
    a. Start my own practice
    b. Join a private practice
        1. What size and type of private practice do you intend to join?
            i. Small (1-4) MD-only practice
            ii. Small (1-4) MD / OD practice
            iii. Medium (5-10) MD-only practice
            iv. Medium (5-10) MD / OD practice
            v. Large (10+) MD-only practice
            vi. Large (10+) MD / OD practice
            vii. Uncertain
    a. Join a hospital-based practice
    b. Uncertain
    c. Other (please specify)

5. Are you currently a single provider or part of a group practice? (Practicing ophthalmologists)
    a. Single provider
    b. Group practice
        1. How many doctors are in your group practice?
            i. MDS
            ii. ODs

6. Which of the following best describes your primary practice setting? (Practicing ophthalmologists)
    a. Private practice
    b. Corporate practice
    c. Private hospital
    d. Public hospital
    e. Institution / military practice
    d. Other (please specify)

7. What is the percent ownership of your practice? (Practicing ophthalmologists)
    a. Self
    b. Other ophthalmologists / optometrists

Copyright © 2023, Market Scope, LLC

c. Private equity
d. Corporate entity

8. How would you describe your ophthalmic focus or specialty? (Practicing ophthalmologists)
    a. Cataract
    b. Refractive
    c. Cataract and refractive
    d. Comprehensive ophthalmology
    e. Glaucoma specialist
    f. Retina specialist
    g. Pediatric
    h. Oculoplastics
    i. Cornea & External Disease
    j. Neuro-ophthalmology
    k. Uveitis
    l. Other (please specify)

9. Which of the following patients do you plan to treat? (Residents)
    a. Cataract
    b. Refractive
    c. Cornea & external disease
    d. Glaucoma
    e. Retina
    f. Pediatric
    g. Oculoplastics
    h. Neuro-ophthalmology
    i. Uveitis
    j. Other (please specify)

Residency Experience

10. Which of the following best describes your perception of your exposure to newer surgical and therapeutic treatments and technologies in residency, as compared to other programs?
    a. I receive(d) more exposure than other residency programs
    b. I receive(d) about the same amount of exposure as other residency programs
    c. I receive(d) less exposure than other residency programs
    d. Uncertain

11. Do you think your experience with technology in residency influenced either the types of technology you use in practice or the quantity? (Practicing ophthalmologists)
    a. Yes
        1. Please describe how your experience with technology in residency influenced how you practice.
    b. No
    c. Uncertain

Copyright © 2023, Market Scope, LLC

12. In general, how likely are you to integrate new technology into your practice in the first year it is available? (Please rate on a scale of 1-5, with 1 being not at all likely and 5 very likely) (Practicing ophthalmologists)

13. How would you rate your residency program regarding training and availability of newer technologies? (Please rate on a scale of 1 to 5, with 1 being very poor and 5 very good) (Residents)

14. How would you rate your residency program's partnerships with industry, in terms of training and collaboration? (Please rate on a scale of 1 to 5, with 1 being very poor and 5 very good) (Residents)

15. Which of the following do you ***currently have/use*** in your practice or OR? (Practicing ophthalmologists)
    a. FLACS
    b. Digital surgical planning software
    c. Image management software / PACS
    d. Heads-up microscope display
    e. Dry eye procedure device (Lipiflow, etc.)
    f. Premium IOLs (multifocal, trifocal, EDOF, toric, post-op adjustable, accommodating, etc.)
        1. How many multifocal IOLs do you typically place per year?
    g. MIGS devices
        1. How many of each of the following do you implant or perform per year?
            i. Hydrus
            ii. XEN Gel
            iii. iStent
            iv. iStent inject/iStent inject W
            v. KDB
            vi. iTrack
            vii. TrabEx
    h. Presbyopia drops
    i. Sustained release drug options / implants
    j. None of the above

16. Which of the following were you trained on, or had access to ***in residency***? (Practicing ophthalmologists)
    a. FLACS
    b. Digital surgical planning software
    c. Image management software / PACS
    d. Heads-up microscope display
    e. Dry eye procedure device (Lipiflow, etc.)
    f. Premium IOLs (multifocal, trifocal, EDOF, toric, post-op adjustable, accommodating, etc.)
        1. Approximately how many multifocal IOLs did you place during residency?
    g. MIGS devices
        1. Approximately how many of each of the following did you implant or perform in residency?

Copyright © 2023, Market Scope, LLC

        i. Hydrus
        ii. XEN Gel
        iii. iStent
        iv. iStent inject/iStent inject W
        v. KDB
        vi. iTrack
        vii. TrabEx
h. Presbyopia drops
i. Sustained release drug options
j. None of the above

17. Which of the following have you ***been trained on, or had access to in residency***? (Residents)
    a. FLACS
    b. Digital surgical planning software
    c. Image management software / PACS
    d. Heads-up microscope display
    e. Dry eye procedure device (Lipiflow, etc.)
    f. Premium IOLs (multifocal, trifocal, EDOF, toric, post-op adjustable, accommodating, etc.)
        1. Have many multifocal IOLs have you placed during residency?
    g. MIGS devices
        1. How many of each of the following have you implanted or performed in residency?
            viii. Hydrus
            ix. XEN Gel
            x. iStent
            xi. iStent inject/iStent inject W
            xii. KDB
            xiii. iTrack
            xiv. TrabEx
    h. Presbyopia drops
    i. Sustained release drug options
    j. None of the above

18. Which of the following do you plan on offering once in practice? (Residents)
    a. FLACS
    b. Digital surgical planning software
    c. Image management software / PACS
    d. Heads-up microscope display
    e. Dry eye procedure device (Lipiflow, etc.)
    f. Premium IOLs (multifocal, trifocal, EDOF, toric, post-op adjustable, accommodating, etc.)
    g. MIGs
        1. Which of the following do you plan on offering?
            i. Hydrus
            ii. XEN Gel
            iii. iStent
            iv. iStent inject/iStent inject W

Copyright © 2023, Market Scope, LLC

v. KDB
vi. iTrack
vii. OMNI
viii. TrabEx
ix. None of the above

h. Presbyopia drops
i. Sustained release drug options / implants
j. None of the above

19. Which of the following best describes the level of discussion/training you receive(d) in your residency program on diversity of brands and manufacturers available for product selection?
    a. Not discussed at all
    b. Minimally discussed but not emphasized
    c. Discussed adequately
    d. Discussed often
    e. Discussed often and highly prioritized

20. Which of the following best describes the level of discussion/training you receive(d) in your residency program on newly developed products on the market (premium IOLs, MIGS, etc.)?
    a. Not discussed at all
    b. Minimally discussed but not emphasized
    c. Discussed adequately
    d. Discussed often
    e. Discussed often and highly prioritized

21. To what degree do you agree or disagree with each of the following statements? (Please rate on a scale of 1 to 5, with 1 being strongly disagree and 5 strongly agree) (Residents)

    I enjoy being trained on newer technology and feel it makes me better prepared for what comes next.

    I prefer to focus on the standard procedures and technology that I am most likely to use in practice to increase my comfort level.

    Having industry partnerships in residency enhances my education and training.

    I am more likely to seek out employment opportunities that value advanced technology or to seek out advanced technology in my own practice because of my exposure in residency.

Copyright © 2023, Market Scope, LLC
